# Supplementary material for: Longitudinal long-read microbiome profiling in a canine model reveals how age, diet, and birth mode shape gut community dynamics
Source: mSystems. 2026 Jan 23;11(2):e01279-25. doi: 10.1128/msystems.01279-25 (PMC12911399; doi:10.1128/msystems.01279-25)
Supplement: Supplemental Figures — Figures S1 to S7. [file msystems.01279-25-s0001.pdf]

A

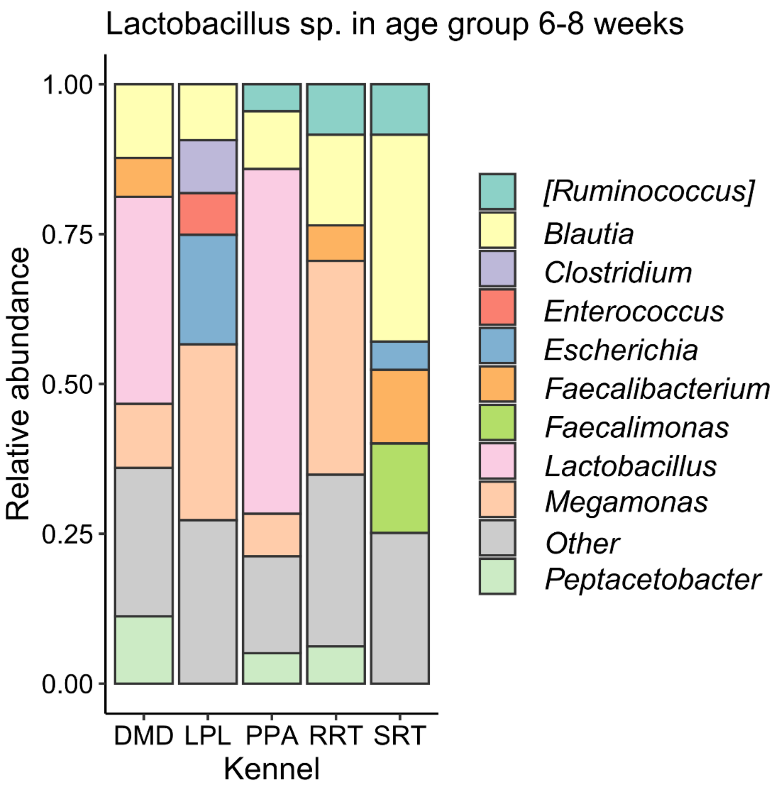

B

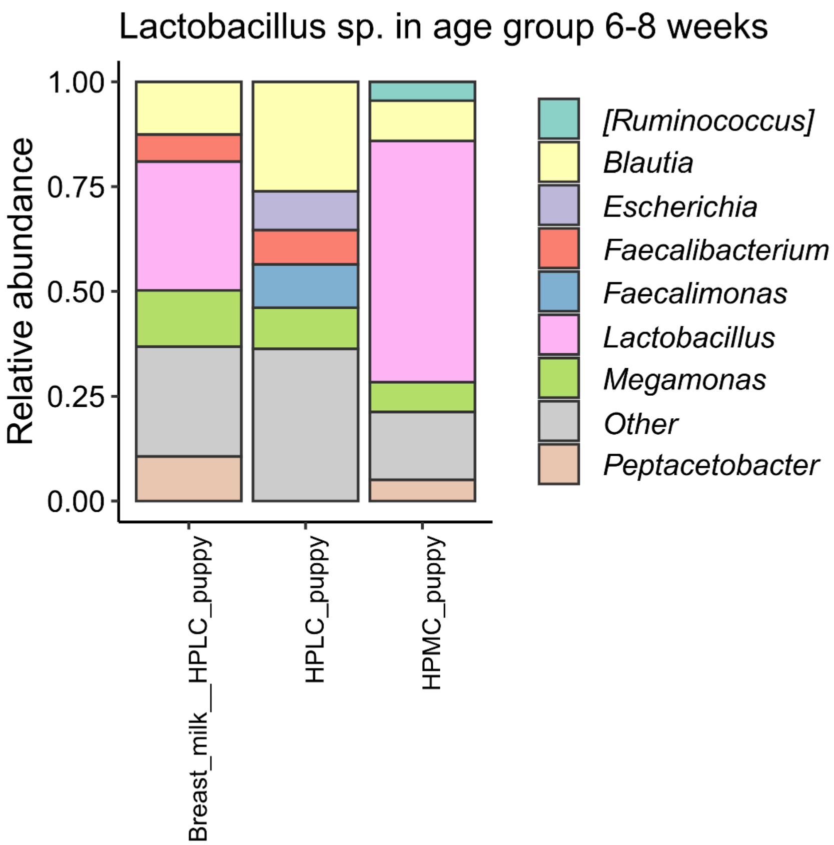

Fig. S1. Early-Weaning Lactobacillus Patterns (6–8 Weeks): Kennel and Diet.
A) Relative abundance of the top 10% most abundant genera, focusing on *Lactobacillus sp.* Presence in the 6-8 weeks age group per kennel. **B)** *Lactobacillus sp.* Abundance per diet in the 6-8 weeks age group.

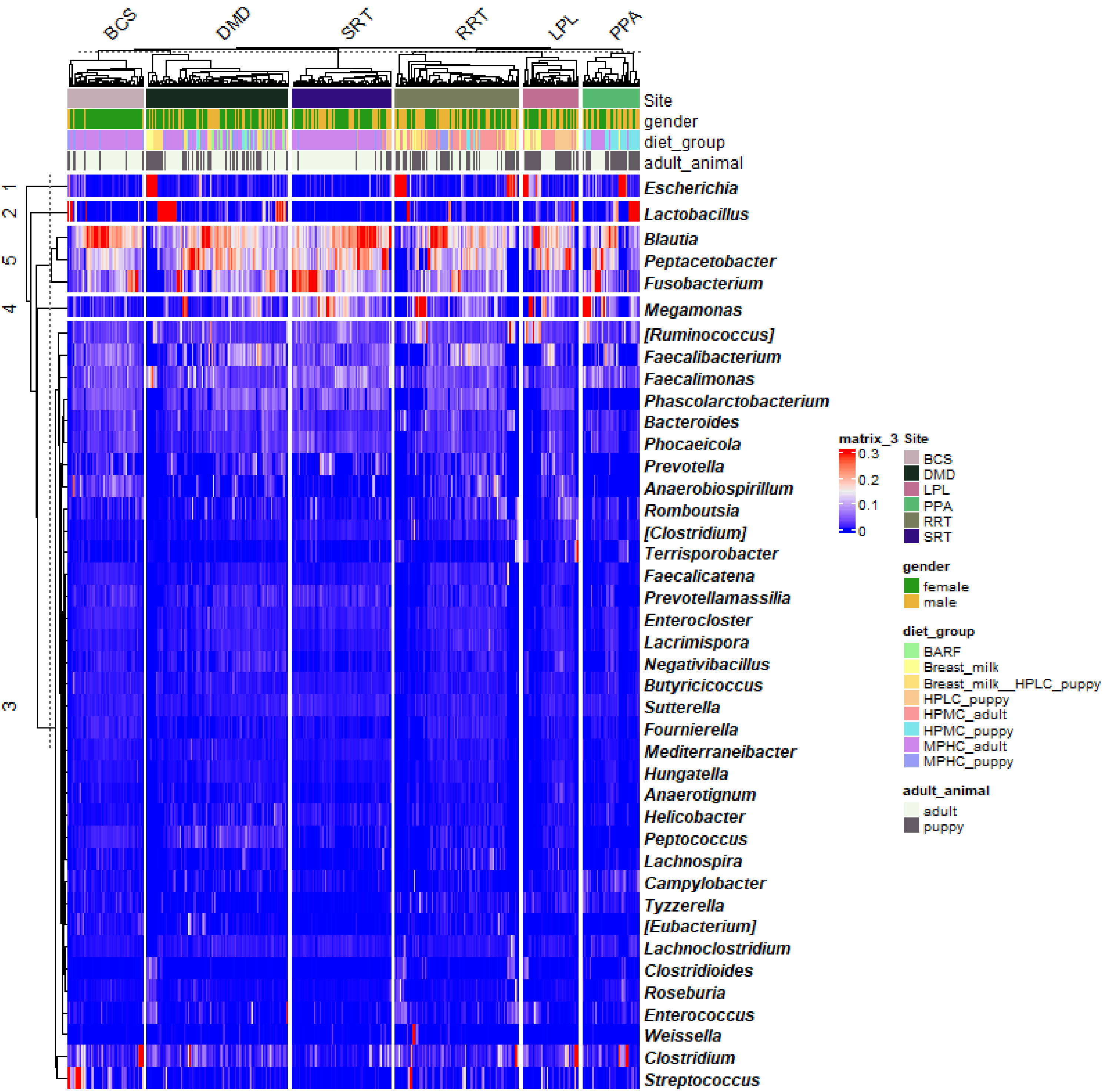

Fig. S2. Heatmap of relative abundances of the top 40 genera across all kennels. Hierarchical clustering shows that age and diet provide more structured clustering than sex. While *Blautia* is represented by several different species in the dataset, complicating the analysis, genus-level clustering results in characteristic patterns for this taxon.

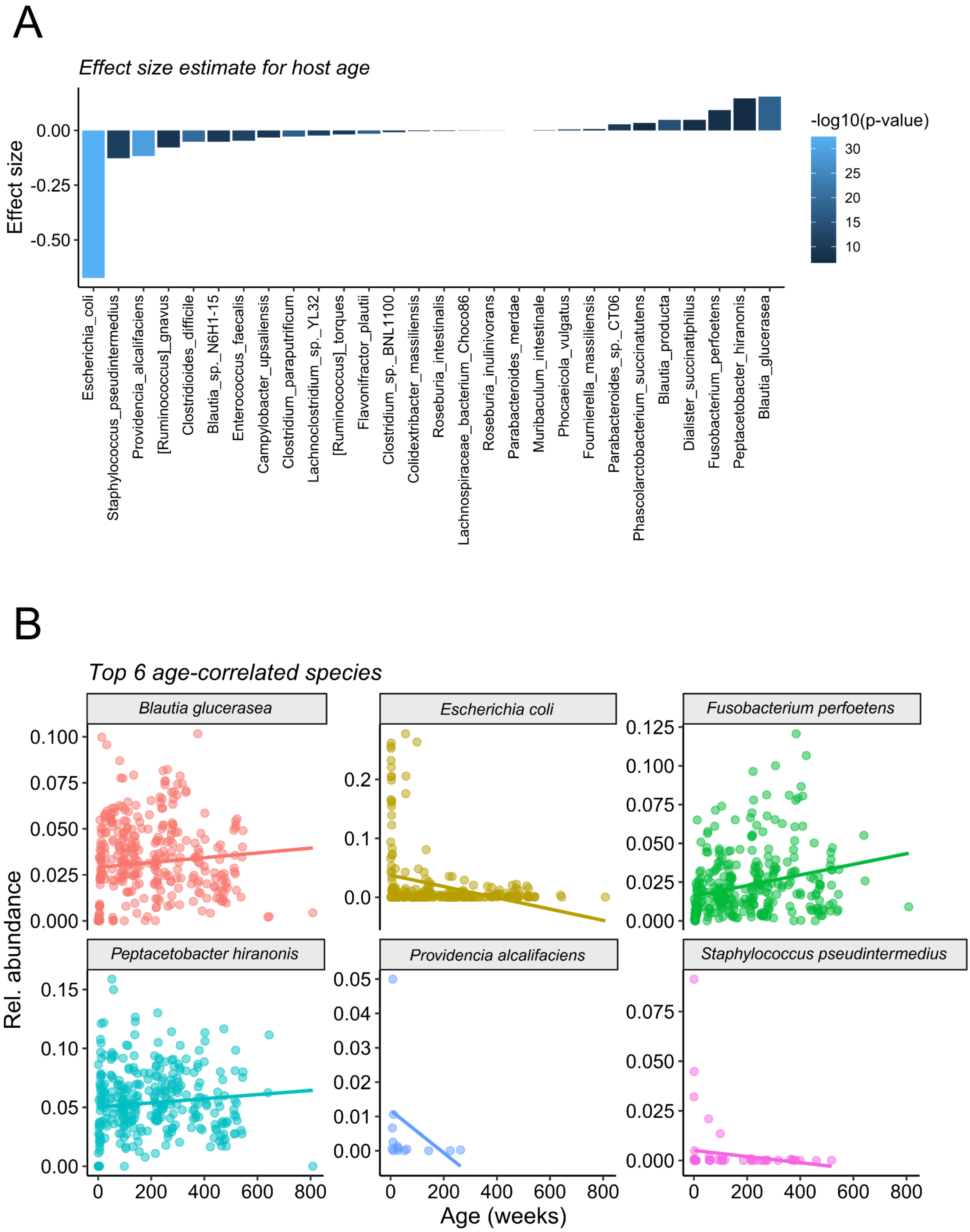

Fig. S3. Age-Associated Species: Effect Sizes and Abundance Trajectories.
A) Bar chart of mixed-effects model estimates for age-correlated species. Y-axis displays the effect size estimate, color gradient is proportional to p-value of the interaction. **B)** Scatter plots of species relative abundance vs. host age in weeks; best-fit lines indicated per species.

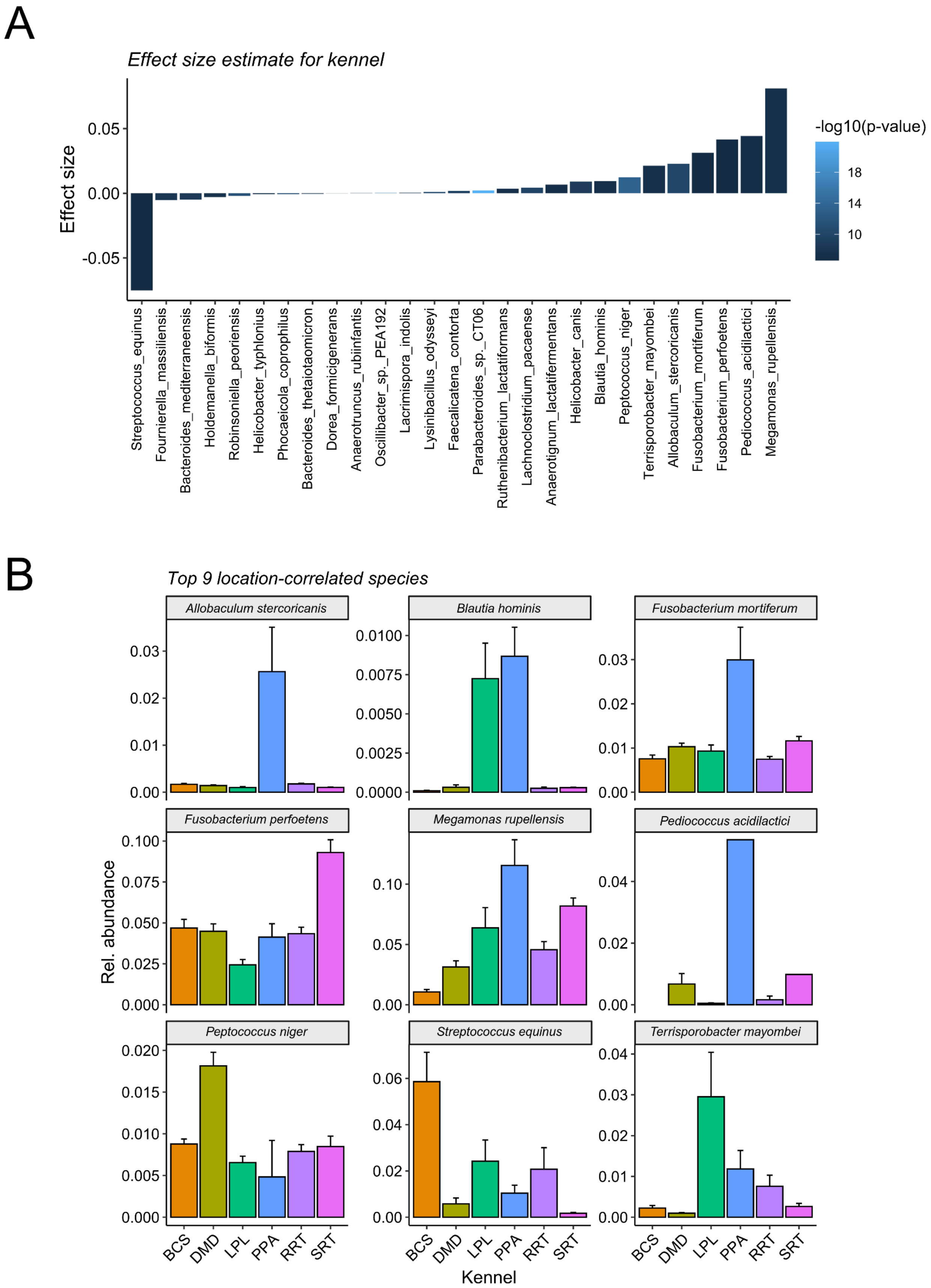

Fig. S4. Kennel-associated microbial composition.
A) Bar chart of mixed-effects model estimates for kennel-correlated species. Y-axis displays the effect size estimate, color gradient is proportional to p-value of the interaction. **B)** Bar charts of species relative abundance vs. location; error bars indicate standard deviation.

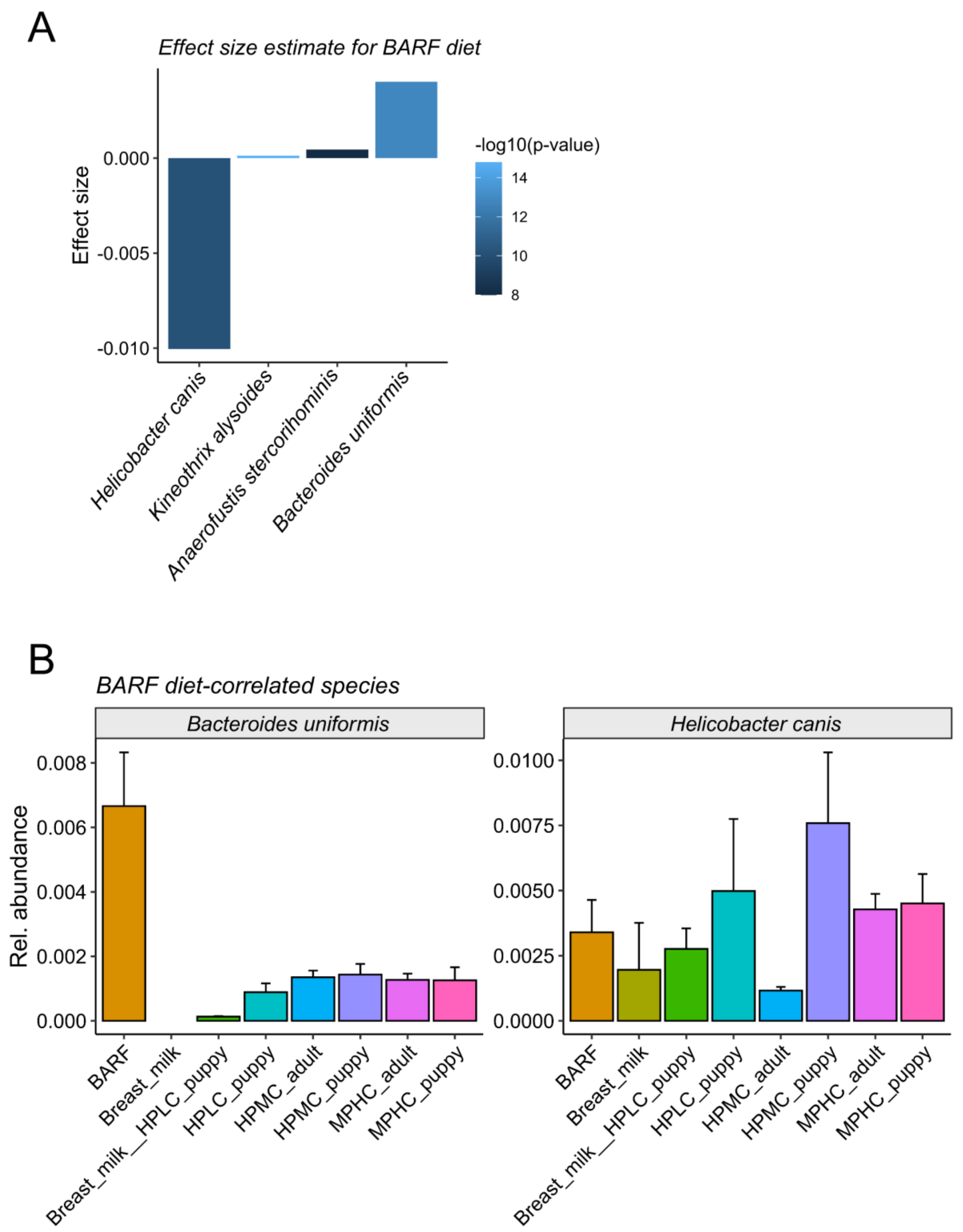

Fig. S5. Effects of BARF-diet on Microbial Composition.
A) Effect size estimates from mixed-effects models for diet-associated species.Y-axis displays the effect size estimate, color gradient is proportional to p-value of the interaction. **B)** Bar charts of species relative abundance vs. Diet category; error bars indicate standard deviation. Abbreviations for host diet terms: HPLC (high-protein–low-carbohydrate), HPMC (high-protein–moderate-carbohydrate), MPHC (moderate-protein–high-carbohydrate).

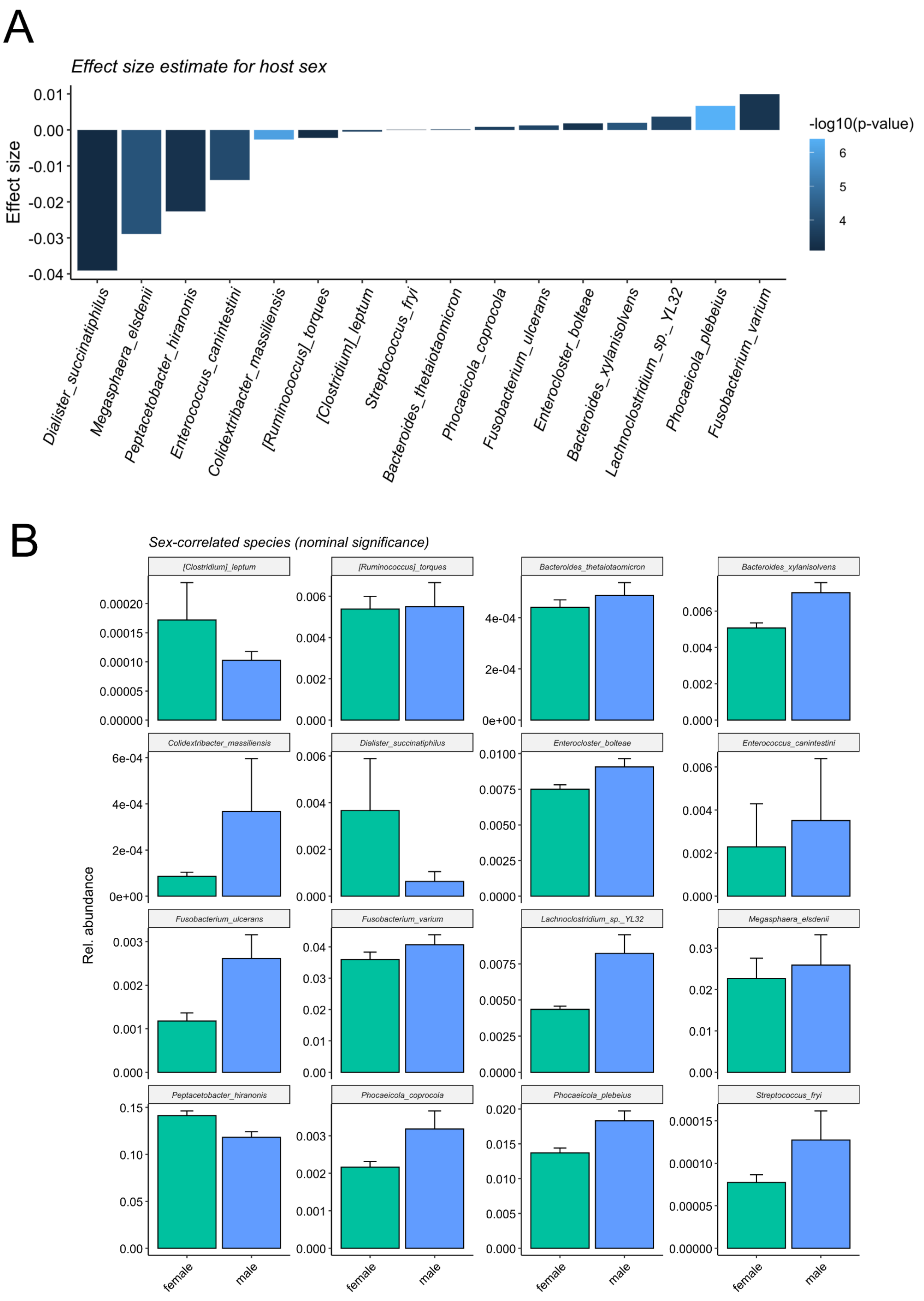

Fig. S6. Sex-Associated Microbial Composition.
A) Bar chart of mixed-effects model estimates for sex-associated species. Y-axis displays the effect size estimate, color gradient is proportional to p-value of the interaction. **B)** Bar charts of species relative abundance vs. sex; error bars indicate standard deviation.

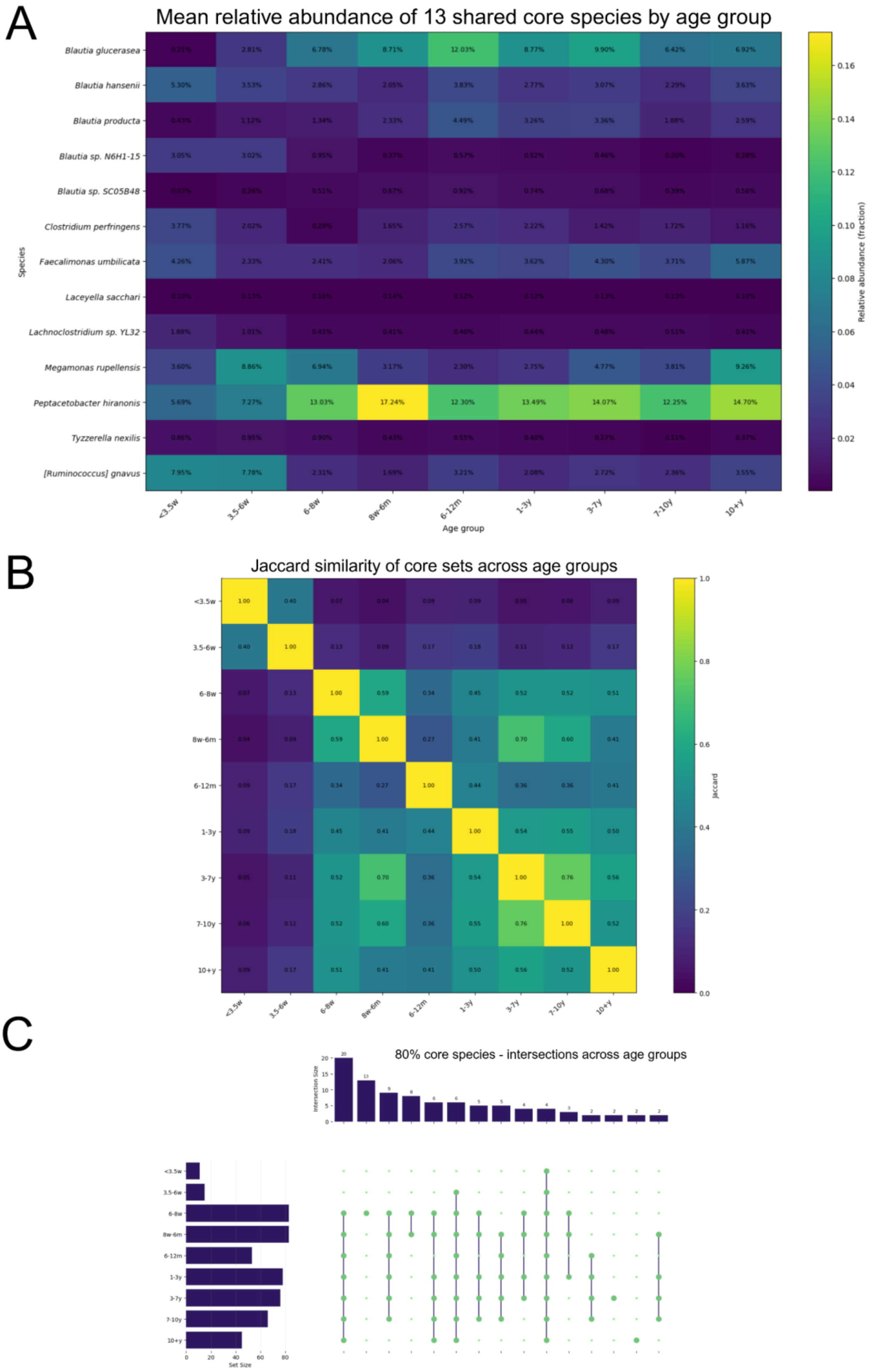

Fig. S7. Species-level core microbiome across age groups.

A) Mean relative abundance (per age group) of the 13 species present in **all** age groups at **≥90% prevalence** (prevalence-only). **B)** Pairwise Jaccard similarity of **90%-prevalence** core sets between age groups. **C)** UpSet plot of **80%-prevalence** core sets: left, set size per age group; top, intersection size; bottom, contributing age groups (green dots, purple connectors). Species labels are italicized at rank. Thresholds and color palette are harmonized across panels as indicated.
